# Gain of function of mutant p53: R282W on the peak?

**DOI:** 10.1038/oncsis.2016.8

**Published:** 2016-02-15

**Authors:** Y Zhang, S V Coillie, J-Y Fang, J Xu

**Affiliations:** 1 State Key Laboratory for Oncogenes and Related Genes, Key Laboratory of Gastroenterology & Hepatology, Ministry of Health, Division of Gastroenterology and Hepatology, Renji Hospital, School of Medicine, Shanghai Jiao Tong University, Shanghai Cancer Institute, Shanghai Institute of Digestive Disease, Shanghai, China; 2 Faculty of Medicine, Catholic University Leuven, Leuven, Belgium

## Abstract

Mutant p53 proteins commonly lose their tumor suppression function and gain novel oncogenic functions (gain of function (GOF)). Different p53 mutations are often considered in one class in biological and clinical studies. However, recent studies have revealed that p53 mutations are biologically and clinically distinct. The R282W mutant associates with earlier onset of familial cancers and poorer outcome of cancer patients, suggesting a more prominent GOF effect of this specific mutant. Here we discuss our current understanding on the multifaceted effects of R282W mutation, including its structural features, signaling pathways and clinical implications. The destabilizing nature, aggregation proneness, altered transcriptome and interactome may collaboratively contribute to the unique phenotype of R282W mutation. The quest for mechanistic insights into the unique GOF effects of R282W mutation would further our understanding of the biology of mutant proteins in cancers, and enforce the development of more effective targeted therapies.

## Introduction

The p53 protein, which is encoded by TP53 gene, plays a pivotal role in the body's anticancer defense mechanisms. However, its function is almost always compromised in tumor cells through gene mutation, deletion, epigenetic silencing or protein degradation.^1^ With the evolvement of the cognition on this protein, a plethora of evidence confirm that mutant p53 proteins not only lose their tumor-suppressive function and acquire dominant-negative activities, but also gain new oncogenic properties that are independent of wild-type p53. Additionally, different mutants exhibit distinct transactivation patterns that are directly connected with disparate phenotypes.^2, 3, 4^ More importantly, studies involving various cancer patients revealed that different p53 mutations were associated with diverse prognostic values.^5^ While hot spots like R273H and R248 have been intensively investigated,^6, 7, 8^ studies concerning R282W have been relatively limited (in this case, R282W designates an arginine mutated to a tryptophan at position 282 in the p53 protein). Of note, we found that the R282W mutant was significantly associated with shorter survival time and earlier onset age of first tumor in the selected Li–Fraumeni syndrome patients, as compared with other hot spot gain-of-function (GOF) mutations.^9^ Studies on non-small-cell cancer patients and chronic myelocytic leukemia patients also attested to its undesirable influence on the development and progression of cancer.^10^ Yet, the cancer-related mechanisms of this mutation are still obscure. To some extent, the R282W loses some wild-type p53 tumor-suppressive activity. On the other hand, it may acquire truly neomorphic or GOF activities that facilitate tumor growth. For a better understanding of mutations on Arg282, here we focus on some current studies on R282W to illuminate the mechanisms of its tumor predisposition.

## Structure

**Figure 1:**
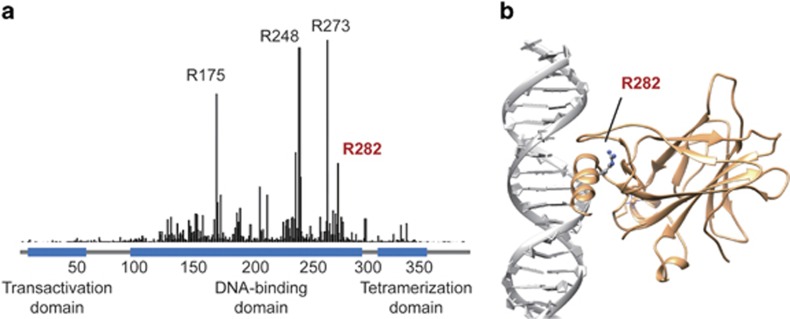
Frequency and the position of R282W mutation. (**a**) The relative frequency of R282W mutation in all human cancers. Data were obtained from the Catalogue of Somatic Mutations in Cancer (COSMIC) database (October 2015 version). (**b**) The structure of p53 DNA-binding domain in complex with a DNA fragment, according to X-ray-determined three-dimensional structure (PDB accession number: 1tup.pdb). The Arg282 residue is located in the H2 helix structural motif.

The p53 protein consists of a transactivation domain (amino acids 1–44), a proline-rich domain (64–92), a central DNA-binding domain (102–292), a tetramerization domain (325–356) and a C-terminal domain (357–393).^11^ The preponderance (95%) of p53 mutations in human cancers are missense mutations, mainly situated within the DNA-binding domain (amino acids 102–292) with hot spots at codons R175, G245, R248, R249, R273 and R282 (Figure 1a).^12, 13^ The DNA-binding domain of p53 protein includes a central *β*-sandwich, which serves as a basic scaffold, and a binding surface with two large loops (L2 and L3) that are stabilized by a zinc ion and a loop–sheet–helix motif. Various p53 response elements make contact to this specific surface.^14^ Arg282 is located in the DNA-binding helix (H2) and packs between the helix and the surface of *β*-strands S2 and S2′ (Figure 1b).^15^ This residue has been classified as the 'structural' residue (like Arg175, Gly245 and Arg249), as it plays a role in maintaining the structural integrity of the DNA-binding surface.^14^

**Figure 2:**
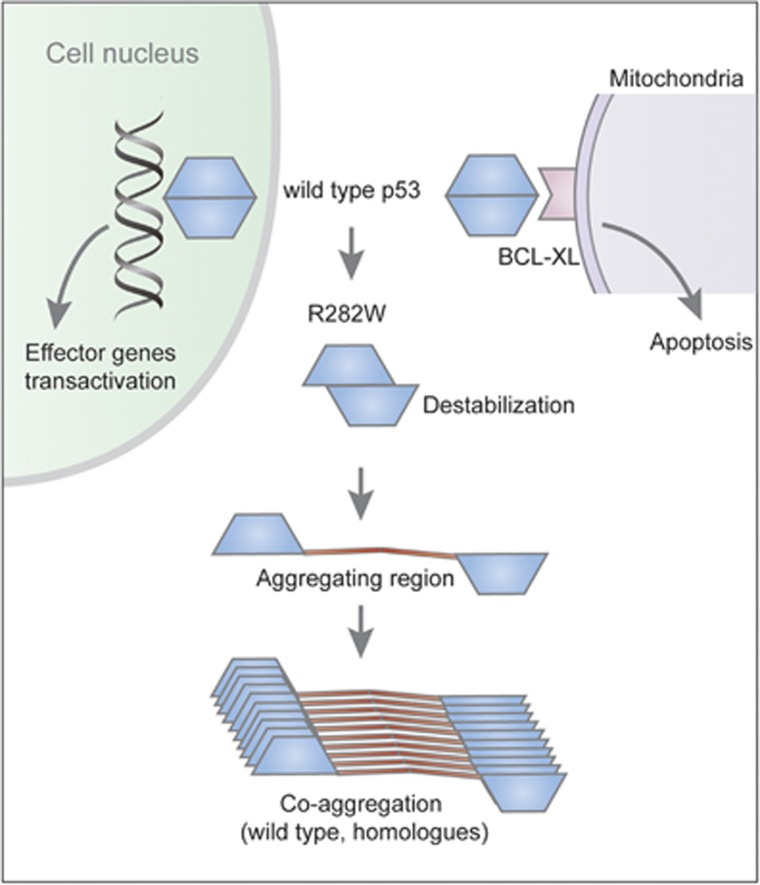
Structural effects of R282W mutation. In response to DNA damage, the wild-type p53 binds to DNA in the nucleus and transactivates downstream effector genes, and it interacts with BCL-XL in the mitochondria and induces mitochondrial apoptosis. The R282W mutation causes destabilization of the protein structure, impairing its binding to DNA and BCL-XL. Moreover, the mutation induces exposure of the aggregation-prone region that is normally buried in the hydrophobic core of p53 protein. This induces coaggregation of the wild-type p53 and its homologues p63 and p73.

The R282W mutation on the H2 helix caused a loss of hydrogen-bond interactions, leading to disruption of the loop–sheet–helix motif.^16, 17^ This structural mutation substantially destabilized the protein thermodynamic stability for up to 3 kcal/mol.^18^ By contrast, a similar effect caused by contact mutations like R248W accounted for <2 kcal/mol, while R273H merely had an impact on the core domain's thermodynamic activity. Besides this, thermodynamic destabilization had severe implications for the folding state of the mutant in the cell (Figure 2). When purified and studied at 20 °C as the isolated core domain, R282W retained 82% of the wild-type DNA-binding affinity. But at 37 °C, R282W was sufficiently destabilized to cause denaturation, leading to the abrogation of normal functioning.^14, 16^ Moreover, in our previous study, R282W mutant exhibited significant aggregation propensity, leading to negative effect on its structure as well as wild-type p53.^19^ However, this mutation–structure and structure–function relationships were still hard to be interpreted. Some proposed that the R282W mutant still retains partial transcriptional ability.^20^

Furthermore, researchers have identified the DNA contact surface of the p53 DNA-binding domain as the binding site for Bcl2 through nuclear magnetic resonance.^21^ Cytoplasmic p53 interacts with Bcl-2 family members to exert their functions, leading to mitochondrial outer membrane permeabilization and apoptosis, either as a direct activator of the Bax/Bak effectors, or as a sensitizer/derepressor of Bcl-x/2 and Mcl1.^22, 23^ Additional studies with nuclear magnetic resonance chemical shift perturbation substantiated that the p53-hot spot mutations R248W, R248Q and R282W were located at the binding surface to Bcl-XL (Figure 2).^9^ These mutations were highly associated with shorter survival period, implying potential relevance of the mitochondrial apoptotic functions of mutant p53 to cancer patient survival. Currently, it is poorly understood whether the R282W mutant may have a distinct protein interactome than other hot spots, and the structural basis of its prominent GOF effects remains unclear.

## Oncogenic mechanisms

Mutant p53 is known to lose its tumor-suppressor activity by exerting an overwhelmingly negative effect on the wild-type allele, serving to alleviate the ability of wild-type p53 to inhibit cellular proliferation and induce apoptosis. Mutant p53 also acquires novel oncogenic functions to regulate phenotypes such as cell growth, migration, invasion, metastasis, genomic instability and chemoresistance.^24, 25, 26^ When it comes to the R282W mutant, current understandings informed that altered protein-interacting and DNA-binding abilities may collaboratively contribute to its unique GOF. Here we discussed them separately.

### R282W interacting with P63 and P73

**Figure 3:**
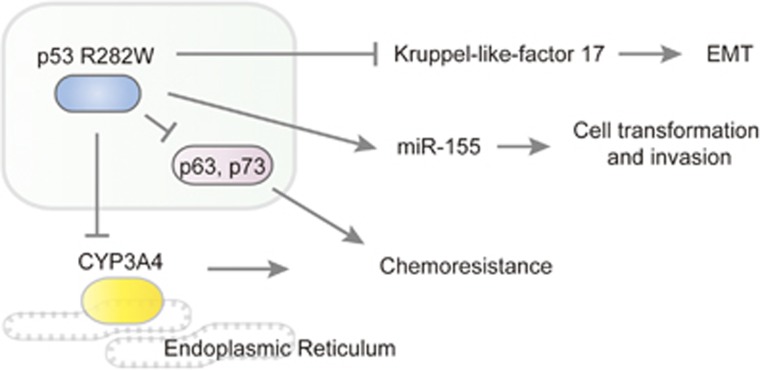
Signaling pathways associated with p53 R282W mutation. The R282W mutant suppresses the expression of KLF17 and thereby induces epithelial mesenchymal transition (EMT). This GOF mutant also induces the expression of miR-155 and promotes cell transformation and invasion. The interaction with p63 and p73 contributes to chemoresistance, and this effect also involves the induction of CYP3A4, which is a member of the cytochrome P450 enzyme responsible for the metabolism of multiple anticancer drugs.

P63 and p73 are members of the p53 gene family, and their transactivation isoforms exhibit certain homology with p53 in modular structure and transcriptional profile. P63 is a master gene for normal epithelial stem cells, protecting them from apoptosis and coordinating their differentiation.^27^ P73 can transcribe endogenous p53-responsive genes such as p21,^28, 29^ and reporters containing various p53-responsive promoters.^30^ Normally, p63 and p73 are rarely mutated in tumors, but they can be inhibited by mutant p53, resulting in enhancement of oncogenic potential of the affected cell lines (Figure 3).^31, 32^ A comprehensive review on p63 as a tumor suppressor and its interaction with mutant p53 has been presented previously.^33^

Chromatin immunoprecipitation (ChIP) analysis in the EI-H1299 cells with inducible p53 R282W demonstrated that mutant p53 may be directly recruited to the promoters of its target genes with p63 (PLK2, DKK1, METTL7B, OCEL1, TMEM205 and TFPI2). These genes were also regulated in R273H and R280K induced cells.^20^ Moreover, it was found that co-expression of p53 mutants (R282W and R110P) with TAp63α/TAp73α drove p63 and p73 to aggregate perinuclearly, while in the presence of wild-type p53, both p63 and p73 were mainly localized in the nucleus.^19^ Together these researches concluded that mutant R282W substantially inhibited p63 and p73 function. The aggregation-associated GOF effect of R282W mutant may also raise the concern whether mouse double minute 2 homolog (MDM2) inhibitors designated to enhance p53 expression may be effective for cancers carrying the R282W mutation.

### R282W binds to Kruppel-like-factor 17 to regulate its function

Kruppel-like-factor 17 (KLF17) is a tumor suppressor transcription factor, which binds to its target gene promoters via CACCC boxes and regulates their expression. KLF17 mainly acts on the promoters of epithelial mesenchymal transition-related genes such as id-1, E-cadherin, ZO-1, vimentin and fibronectin to inhibit them.^34^ Studies in metastatic breast cancer cells drew the conclusion that by either directly or indirectly binding to the promoter of KLF17, mutant p53-R282W decreases the metastasis suppressor function of KLF17 in these cell lines to facilitate cancer progression.^35^ Similar results can be observed in other mutants such as R175H-, R273H- and R280K-induced breast cancer cell lines (Figure 3). These findings suggest that mutant p53 could inhibit the transcription of genes as well as transactivate other genes.

### R282W-regulated protein-coding genes

CYP3A4 is part of a cluster of cytochrome P450 genes on chromosome 7q21.1, and encodes a member of the cytochrome P450 superfamily of enzymes, which are monooxygenases that catalyze many reactions involved in drug metabolism and synthesis of cholesterol, steroids and other lipids. The CYP3A4 protein is localized in the endoplasmic reticulum, of which the expression is precipitated by glucocorticoids and some pharmacological agents. This enzyme is involved in the metabolism of a majority of drugs in use today, including FOLFIRI (folinic acid/fluorouracil/irinotecan) regimen, etoposide, rapamycin and other antineoplastic drugs (Figure 3).

Data analysis on gene set enrichment analysis revealed that p53 R248 and R282 mutations significantly upregulate CYP3A4 mRNA and protein levels, and cancer cell lines bearing these two p53 mutations displayed resistance effects to several CYP3A4-metabolized chemotherapeutic drugs.^36^ These results ascertained this protein-coding gene as a downstream factor of these two mutants' regulatory profile. Several mechanisms may be involved in the regulation of CYP3A4 by mutant R282W, such as binding directly/indirectly to the promoter of CYP3A4 gene or promoting its protein stability by upregulation of molecular chaperones.^37^

### R282W-regulated noncoding RNA genes

Noncoding RNA is commonly recognized as RNA that does not encode a protein, including microRNAs, small nuclear RNAs as well as other classes. These RNAs regulate various levels of gene expression in physiology and development, and play a significant role in cancer.^38^ MicroRNAs comprise 20–24-nucleotide-long RNAs that are involved in the posttranscriptional control of gene expression. By binding to target mRNAs through their 3′ untranslated regions and recruiting the RNA-induced silencing complex, these RNAs mediate the inhibition of translation and the degradation of the respective mRNA.^39^

An *in vitro* study discovered that induced expression of either p53 mutant R248Q or R282W in the p53-null H1299 background was associated with a dose-dependent increase in MIR155HG (the precursor transcript for miR-155) expression or mature miR-155 levels, which promoted cellular transformation and invasion. Furthermore, genes including ZNF652, PDCD4, TCF12 and IL17RB were corroborated as critical targets of the mutant p53–miR–155 axis in breast cancer (Figure 3).^40^ To date, no microRNAs genes have been affirmed to be directly regulated by mutant R282W.

From the above, it seems that mutant R282W does not function in an exclusive profile. In addition, data analysis on gene profiling indicated that R175H, R248Q, R248W, R249S, R273H and R282W regulated a partially overlapping gene set.^20^ Further hierarchical clustering of the expression profiles for the hot spot p53 mutants revealed that R282W shared the most common genes with R248W, which was in line with the results of gene set enrichment analysis performed by another study.^9^ Although R282W and R248W mutations belong to 'structural' and 'contact' mutations, respectively, they are both situated on the binding surface to BCL-XL, as suggested previously.^36^

## R282 mutation and early onset of familial cancers

Study on a p53 germline mutation database via a multivariate COX regression model suggested that mutation of R282 is related to a significantly earlier onset age of first tumor in the selected Li–Fraumeni syndrome patients than the nonsense (loss-of-function) mutations, while mutations on G245 residue showed later cancer-onset age. Data analysis also showed out that R282 mutation was more frequently detected in tumors of the bone, while the R175 and R248 were enriched in brain tumors, inferring that mutant p53 can lead to different types and strengths of GOF effects.^36^

## R282W and cancer prognosis

Kaplan–Meier survival analysis on the Memorial Sloan Kettering Cancer Center (MSKCC) bladder cancer data set^41^ in combination with, The Cancer Genome Atlas^42^ patient cohorts verified that patients carrying p53 mutations on Arg248 and Arg282 residues had shorter overall survival time than those carrying nonsense mutations.^9^ In addition, this result was confirmed by multivariate survival analysis on an independent data set extracted from published literature.^9^ Besides, some *in vitro* studies found that p53 mutation at codon 282 was radio-resistant, albeit this phenomenon is not exclusive to the R282 mutant.^43, 44^ Based on these findings, it is worthy to further study whether p53 hot spot mutations may represent distinct biomarkers for cancer prognosis.

## Conclusions

In conjunction with the findings above, the R282W mutant exhibits certain extents of GOF and is highly associated with the clinical prognosis of patients with cancer. Future studies to elaborate the detailed regulatory microRNA, mRNA and protein-related mechanisms through which the R282W mutant promotes GOF phenotypes will be significant for the development of novel cancer therapeutics.
